# Salt Dependence of the Tribological Properties of a Surface-Grafted Weak Polycation in Aqueous Solution

**DOI:** 10.1007/s11249-017-0963-0

**Published:** 2017-12-05

**Authors:** Maryam Raftari, Zhenyu J. Zhang, Steven R. Carter, Graham J. Leggett, Mark Geoghegan

**Affiliations:** 1 Department of Physics and Astronomy, University of Sheffield, Sheffield, S3 7RH UK; 2 Department of Chemistry, University of Sheffield, Sheffield, S3 7HF UK; 3 Present Address: School of Chemical Engineering, University of Birmingham, Birmingham, B15 2TT UK

**Keywords:** Contact mechanics, Polymer films, Polyelectrolytes, Atomic force microscopy, Adhesion

## Abstract

The nanoscopic adhesive and frictional behaviour of end-grafted poly[2-(dimethyl amino)ethyl methacrylate] (PDMAEMA) films (brushes) in contact with gold- or PDMAEMA-coated atomic force microscope tips in potassium halide solutions with different concentrations up to 300 mM is a strong function of salt concentration. The conformation of the polymers in the brush layer is sensitive to salt concentration, which leads to large changes in adhesive forces and the contact mechanics at the tip–sample contact, with swollen brushes (which occur at low salt concentrations) yielding large areas of contact and friction–load plots that fit JKR behaviour, while collapsed brushes (which occur at high salt concentrations) yield sliding dominated by ploughing, with conformations in between fitting DMT mechanics. The relative effect of the different anions follows the Hofmeister series, with I^−^ collapsing the brushes more than Br^−^ and Cl^−^ for the same salt concentration.

## Introduction

Polymers are used to control friction in numerous applications. When high friction is required, it is possible to use low-modulus polymer materials with increased surface roughness [1]. Controlling texture is optimized in many commercial applications, such as tyres [2]. However, there are other routes to increase friction where stiff surfaces are required [3]. When lubrication is necessary, grafting polymers on surfaces may be applied for both oil-based [4–6] and aqueous [6–9] environments. Such grafted polymers are known as brushes [10] and are often used for modifying surfaces to improve material compatibility, to prevent aggregation (steric stabilization), or to add functionality.

When polyelectrolyte brushes are fully solvated in aqueous environments, osmotic pressure within them and steric repulsion upon compression contribute to their exceptional lubrication characteristics. Their solvation changes as a function of environment such as pH, and this in turn affects lubricity [11–13]. The salt-dependent frictional behaviour of polyelectrolytes has been studied for two gels in contact with each other [14] and for strong polyelectrolyte brushes [15, 16]. There is a tension between the pH and salt dependence of polyelectrolytes, since pH is often considered to convey “smart” properties to the material, with an aim of triggering an environmental stimulus, e.g. for adhesion [17–20] and drug delivery [21, 22]. The addition of salt screens ions and consequently reduces the effect of pH, which is particularly important in physiological environments. It is therefore important to fully understand the effects of salt on polyelectrolyte properties, including those of friction. Polyelectrolytes are candidates for use in biomedical devices and implants due to their inherent material processing versatility [21, 23]. Although friction is often considered an engineering matter, its importance in biomedical implants cannot be understated, where pressures of several MPa are exerted on moving joints [24].

The addition of salt to weak polyelectrolyte brushes (those for which the charge can be readily controlled by pH) hides a deceptively rich physics [25, 26]. For thin charged brushes in salt-free solution, a double layer is formed with a layer of oppositely charged counter-ions adjacent to the brush. For thicker brushes, the counter-ions can penetrate the brush, which is swollen by these (osmotic swelling) rather than Coulombic repulsion along the chain. As more salt is added, there is initially an increase in osmotic swelling, but for greater concentrations of salt, the charges are shielded and the swelling is reduced. At a salt concentration similar to that of the free counter-ions within the brush, the brush is considered “salted”, and there is considerably less swelling than in the salt-free environment [27, 28].

Salt concentration, pH, and polyelectrolyte molar mass and grafting density determine the swollen state of polymer brush, which consequently affects the tribological behaviour of the surface. A consideration of the contact mechanics of these surfaces involves the mechanical properties of the brush and the probe interrogating it. For contact at the nanoscopic scale, the problem can be limited to a choice between a Johnson–Kendall–Roberts (JKR) [29] approach for deformable elastic materials or the Derjaguin, Muller, and Toporov (DMT) model for more rigid materials [30]. The two models are compatible with each other insofar as it is possible to consider interactions that are in between the JKR and DMT models [31] and that in the absence of any surface adhesion (*γ* = 0) both models reduce to that of Hertz [32]. In the JKR model, the contact area is given by

1 $$ A = \uppi \left( {\frac{R}{K}\left( {N + 3\uppi \gamma R{ + }\sqrt {6\uppi \gamma RN + \left( {3\uppi \gamma R} \right)^{2} } } \right)} \right)^{2/3} , $$

where *R* is the radius of the probe, *K* is the elastic modulus of the surface being interrogated (assuming a non-deformable probe), *γ* is the thermodynamic energy of adhesion, and *N* is the applied load. For the DMT model,

2 $$ A = \uppi \left( {\frac{R}{K}} \right)^{2/3} \left( {N + 4\uppi \gamma R} \right)^{2/3} . $$

The JKR model neglects any adhesive forces originating outside the area of contact which gives rise to a singularity in stress at the contact. On the other hand, the DMT approach treats the contact profile at the interface as Hertzian even though adhesion forces outside the contact zone are considered. Rationalizing the two models with their unsatisfactory characteristics has been a focus of substantial research with the goal being a unifying parameter, the value of which would dictate which of the two situations was applicable. In both the JKR and DMT approaches, the force required to separate the two surfaces, the “pull-off” force, is independent of material elastic constants, being given by

3 $$ N_{\text{PO}} = \lambda \uppi \gamma R , $$

where *λ* = 3/2 and 2 for the JKR and DMT models, respectively. Reconciling these differences requires an understanding of the material properties of the probe and the surface and a suitable means was found to achieve this first in a simple theory [33], which has subsequently been improved numerically [34, 35]. A so-called Tabor parameter, which is given by

4 $$ \mu = \left( {\frac{{R\gamma^{2} }}{{K^{2} \varepsilon }}} \right)^{{\frac{1}{3}}} , $$

where *ε* is a constant of the order of ~ 0.5 nm, has extended our understanding of contact mechanics, but its application to experimental results is difficult because these are often realized by a contact diameter which is not readily extracted from this theory. Another approach consolidated both of these models through the use of a transition parameter, *α*, which is related to the contact radius, *a* (where *A* = π*a* ^2^) by

5 $$ a = a_{0} \left( {\frac{{\alpha + \sqrt {1 - N/N_{\text{PO}} } }}{1 + \alpha }} \right)^{2/3} , $$

where the “pull-off” force is here the force required to separate the AFM tip from the surface. This Eq. (5) is known as the general transition equation.

The single asperity contact mechanics of polymer brush layers is ideally studied by lateral or friction force microscopy (FFM) [36–38], a technique in which an atomic force microscopy (AFM) tip of radius typically between 10 and 50 nm is dragged over a surface and the frictional force is monitored as a function of applied load. The technique is important because it combines a small contact area due to the AFM tip and the ability to accurately measure sub-nN forces.

Recent work has shown that there is a low adhesion regime whereby single asperity contact mechanics is insufficient to describe the interaction of a polymer brush with an AFM tip [11, 12, 39–44]. The AFM tip can reasonably be considered as a single asperity probe given its small radius. However, the tip can also penetrate the brush, deforming it and dissipating energy in the process. This has been termed “ploughing” [31, 36, 45], and experiments on self-assembled monolayers have shown that these interactions can be treated as a combination of a load-dependent term (which takes the same form as the macroscopic Amontons’ law) [46] and a term reflecting the stress required to retain a sliding contact between the tip and the surface [47–49]. This yields

6 $$ F = \mu (N + N_{\text{PO}} ) + \tau \uppi \left( {\frac{{R (N + N_{\text{PO}} )}}{K}} \right)^{2/3} , $$

where *F* is the lateral force on the tip, *τ* is a shear strength, and *μ* is the friction coefficient.

Model polymer brushes were provided using poly[2-(dimethylamino)ethyl methacrylate] (PDMAEMA), which is a weak polycation. Previous work on this polymer shows that it swells slightly on the addition of a small amount of salt, but that its thickness is reduced at larger concentrations [50]. Atomic force microscopy-based techniques have also demonstrated the conformational collapse of PDMAEMA brushes at high salt concentration [51, 52]. In this work, the frictional behaviour of brushes of PDMAEMA in contact with AFM tips coated with gold or a PDMAEMA grafted layer has been measured as a function of the concentration of solutions of different monovalent potassium salts: KCl, KBr, and KI, which allow the effect of the specific anion to be tested. It is shown that the size of the anion has a critical role in both the conformational and tribological properties of the brush.

## Experimental

### Materials

Silicon wafers (boron doped, 0–100 Ω cm, and (100) orientation) were purchased from Prolog Semicor (Ukraine). Copper(I) chloride (99.999%), copper(II) bromide (99.999%), [11-(2-bromo-2-methyl)propionyloxy]undecyl trichlorosilane, dry toluene (99.8%), 2-(dimethyl amino)ethyl methacrylate (C_8_H_15_NO_2_), KCl (99%), KI (99%), and KBr (99%) were all purchased from Aldrich and used as received. HPLC-grade acetone and methanol were purchased from Fisher Scientific, and 2,2′-dipyridyl (99%) was purchased from Acros. Non-conductive silicon nitride triangular probes (MLCT, from Brucker) with nominal spring constant 0.065 Nm^−1^ and radius 20 nm were used for the FFM experiments.

### Brush Synthesis and Modification of the AFM Cantilever

PDMAEMA brushes were grafted from silicon substrates and AFM tips by atom transfer radical polymerization (ATRP), using the same method as in earlier experiments [11, 12]. To immobilize the initiator, a clean silicon substrate and AFM tip were immersed for 6 h in 20 mL of dry toluene solution containing 50 μL [11-(2-bromo-2-methyl)propionyloxy]undecyl trichlorosilane (initiator). When coated, the substrates and AFM tip were rinsed with toluene, and then dried under nitrogen gas. 2,2′-Dipyridyl (0.225 g), CuCl (0.0624 g), and CuBr_2_ (0.0084 g) were added together as catalysts. These catalysts were dissolved by adding degassed acetone (15.9 mL) and 1.5 mL deionized (DI) water. The ATRP monomer solution was completed by adding the 10.8 mL 2-(dimethyl amino)ethyl methacrylate (DMAEMA) to the catalyst solution. Finally, 20 mL of the ATRP solution was injected into a cell (sealed under nitrogen), which contained the initiator-coated silicon wafer and AFM tip. The PDMAEMA film and the PDMAEMA-coated AFM tip were removed and rinsed with methanol after 16 h.

Gold-coated cantilevers were prepared using an Edwards Auto 306 bell jar vacuum coater system. First, a 2-nm-thick adhesion layer of chromium was deposited on the cantilever at a rate of less than 0.05 nm/s. This was then coated with 10 nm of gold at a rate of 0.03 nm/s.

### Brush Characterization

**Fig. 1:**
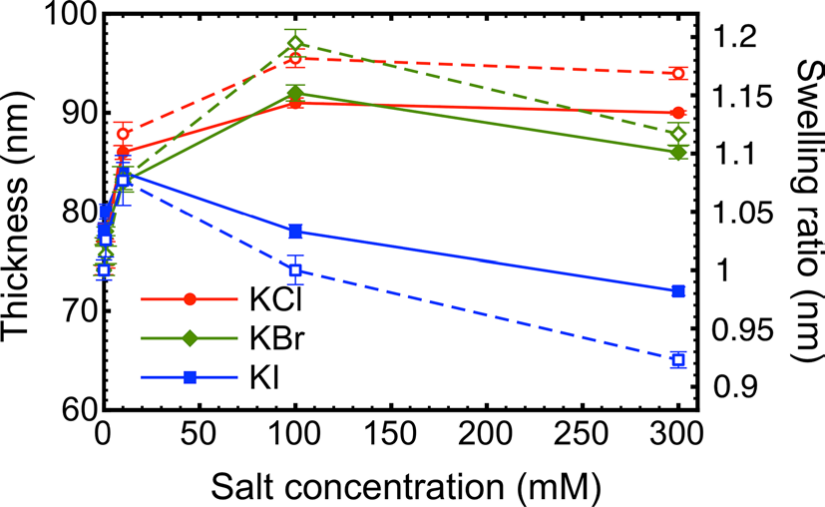
Ellipsometric thickness and swelling ratio of PDMAEMA brushes in different salt solutions. Unfilled symbols correspond to the swelling, filled symbols to the thickness. Error bars may be obscured by the relevant datum. Solid lines are guides for the eye

The average thickness of the PDMAEMA brush was measured using a Woollam M2000V rotating compensator ellipsometer in a dry environment, in deionized water, and in different salt solutions with concentrations ranging from 1 to 300 mM. Ellipsometry measurements were taken using wavelengths from 200 to 1000 nm, and the data were fitted using the analysis software WVASE32 (J. A. Woollam). In order to measure the thickness of the PDMAEMA brush in the solutions, first the sample was placed in the PTFE liquid cell and then 20 mL DI water was added to the cell. The sample was then left in DI water for 10 min before the ellipsometry measurements were taken to allow the thickness to equilibrate. The brush thickness was then determined as a function of the concentration of the different types of salt (Fig. 1). The ratio of the thickness to the dry brush thickness (swelling ratio) is also shown in Fig. 1. To check the thickness of PDMAEMA brushes on the cantilever, a Carl Zeiss 1540XB scanning electron microscope (SEM) was used to take images from a brush-modified cantilever. Free (i.e. not grafted) PDMAEMA was synthesized and characterized by gel permeation chromatography, from which a molar mass of 39 kg/mol was determined. The grafting density of the PDMAEMA was thus determined to be 0.84 chains/nm^2^ [12].

### Friction Force Microscopy

A Digital Instruments Nanoscope IIIa Multimode atomic force microscope was used for friction force measurements operating in contact mode with a liquid cell/tip holder. The AFM probes were non-conductive silicon nitride triangular probes (MLCT, Bruker, California, USA) with extremely low spring constants for high force sensitivity. These silicon nitride probes were modified with a PDMAEMA brush or coated with a thin layer (10 nm) of gold as described above. FFM measurements were taken at a scan rate of 0.99 Hz with 256 points per line and with a scan area of 1 μm × 1 μm. The spring constants of PDMAEMA-coated and gold-coated cantilevers were calibrated by a Digital Instruments PicoForce module and its associated software, based on the method of Hutter and Bechhoeffer [53]. The spring constants were determined to be 0.073 and 0.068 Nm^−1^ for the PDMAEMA- and gold-coated tips, respectively. The optical lever sensitivity of each modified cantilevers was calibrated in DI water before each set of experiments. The friction force was acquired by converting the lateral signal collected by the photodetector from voltage to newton using the wedge method [54, 55], where the cantilever is scanned across a calibration grating (TGF11, MikroMasch, Tallinn, Estonia) and the frictional signal is measured as a function of applied load.

The frictional behaviour between the PDMAEMA brush and each AFM-coated tip was measured in deionized water and with three different monovalent salt solutions with different anions including potassium chloride (KCl), potassium bromide (KBr), and potassium iodide (KI) at salt concentrations from 0 to 300 mM.

## Results

### Brush Thickness

The swollen state of the PDMAEMA brush, as reflected by the measured thickness, is shown as a function of salt concentration in Fig. 1. For KCl and KBr, the brush thickness increases with increasing salt concentration up to 100 mM. For KBr, a small reduction in thickness is observed from 100 to 300 mM, while for KCl the thicknesses measured at 100 mM and 300 mM are similar. For KI, the thickness increases initially (up to 10 mM), but thereafter decreases significantly at higher salt concentrations. The reduction in thickness of the brush in KI indicates that it is salted, which occurs at salt concentrations in excess of that of the counter-ions [56]. The anion dependence of the thickness is consistent with the results of a study of Hofmeister ions on a strong polycation [57].

The increase in brush thickness at low salt concentrations is due to the extra osmotic swelling provided by the additional ions in the solution. However, at higher concentrations screening negates this effect and the interaction between the anions and the amine functional groups becomes important. It has been suggested that the total amount of aqueous hydrogen bonding decreases with decreasing ion size [58], which means that larger ions have a greater affinity for the amine group on the PDMAEMA brush and can consequently more effectively screen the charge of the polymer chain.

### Adhesion

**Fig. 2:**
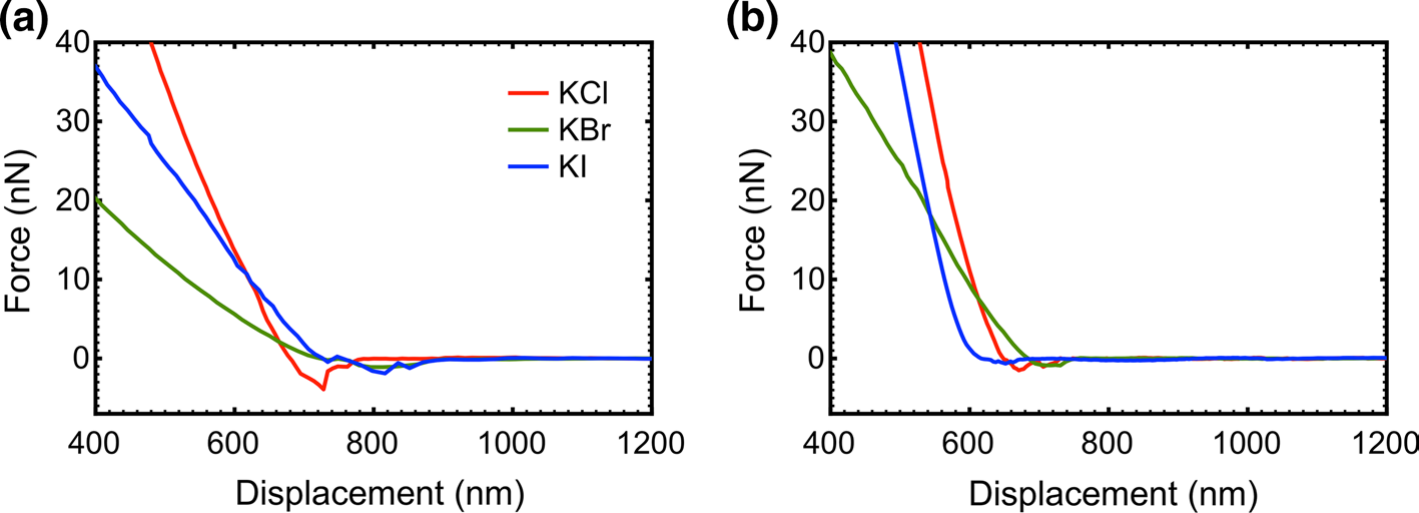
Retraction curves for the **a** gold-coated and **b** PDMAEMA brush-coated AFM tips from the PDMAEMA brush in different 300 mM salt solutions. The different slopes for the curves at small displacements indicate a different laser alignment of the sample

Adhesive interactions between surface-grafted PDMAEMA brushes and gold- or PDMAEMA brush-coated probes were measured by retracting the AFM tip from the substrate in different salt solutions. These data were obtained from 100 measurements. Figure 2a shows retraction curves between gold-coated probes and the PDMAEMA brush samples immersed in different monovalent salt solutions with 300 mM concentration. In Fig. 2b, curves are shown for the retraction of the PDMAEMA-coated tip from the brush. The force of adhesion is the difference between the baseline (i.e. the force at large distances, which can be considered zero) and the minimum force of the curve. This is herein referred to as the pull-off force.

**Fig. 3:**
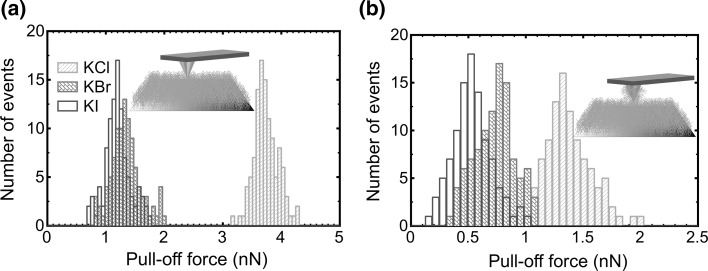
Histograms showing the number of events as a function of the pull-off force from the PDMAEMA brush in solutions of different salts for the **a** gold-coated and **b** PDMAEMA brush-coated AFM tips, as schematized in the insets. The salt concentration in each case was 300 mM

**Fig. 4:**
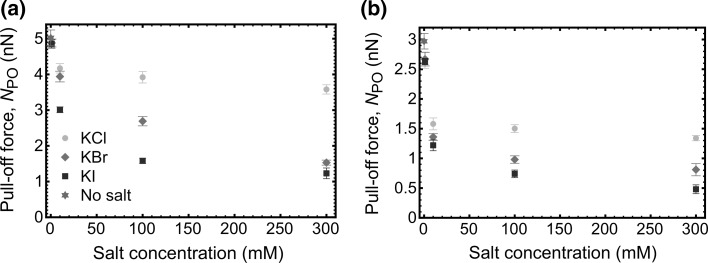
Pull-off force, *N*_PO_ from the PDMAEMA brush for the **a** gold-coated and **b** PDMAEMA brush-coated AFM tips as a function of salt concentration for the three different salts used. Error bars may be obscured by the relevant datum

Histograms (Fig. 3) of the pull-off force distributions for samples in different salt solutions at 300 mM salt concentration show that, for gold-coated probes, adhesion is strongest in KCl solution but is significantly reduced in solutions of KBr and KI. For PDMAEMA-coated probes, the strength of adhesion is reduced in all three salt solutions. However, the magnitude of the reduction is greatest for KCl solutions. The pull-off forces acquired in the three different types of salt are shown as a function of salt concentration in Fig. 4 for the two different tip coatings. It was found that the pull-off force decreases with increasing ionic strength for all salts examined. However, the decrease in the adhesion force is different for each salt; for example, the adhesion force decreases rapidly with increasing concentration of KI, and this is slower in KBr solution. In KCl solutions, the change is comparatively modest for both types of probe. Such a reduction is likely to be due to the binding of anions to the amine functional groups, which consequently screen the electrostatic interactions and reduce attractive interactions. These results are consistent with the ellipsometry data, where the binding of anions is important at larger ion concentrations. This is best explained by a change in the solvation state of the polymer. Swollen brushes bind large amounts of water and have small elastic moduli; they are thus readily deformable and while the thermodynamic work of adhesion (the work done in separating unit area of the tip–brush contact) is small, the contact area is large so that the net adhesion force is greater. In contrast, once the polymer takes a less extended conformation, the elastic modulus of the brush layer increases and the tip–sample contact area decreases. Although the thermodynamic work of adhesion is larger, the net adhesion force is reduced.

### Friction

**Fig. 5:**
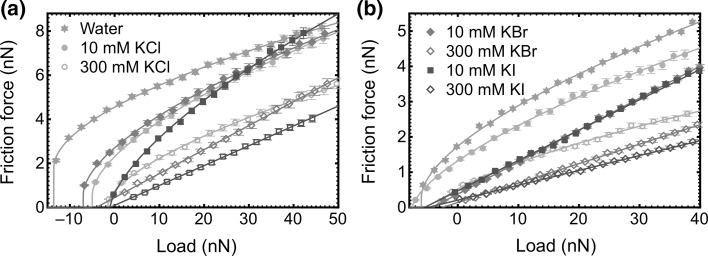
Friction–load curves for the **a** gold-coated and **b** PDMAEMA brush-coated AFM tips from the PDMAEMA brush. The legend is spread across both graphs

To understand the contact mechanics of the PDMAEMA brush, friction–load plots for the two different tip coatings are shown in Fig. 5. A sub-linear relationship between frictional force and applied load was found for measurements acquired at low salt concentration, suggesting a large contact area between the AFM tip and the brush layer. However, at greater salt concentrations for heavier ions, the relationship becomes linear suggesting a change in the conformation of the brush layer [46]. Because there was no material loss between measurements, the transition from a non-linear to linear relationship can only be attributed to the solvation state of the brush. This phenomenon has been reported for a zwitterionic polymer brush that was exposed to solvents of different quality [39].

**Fig. 6:**
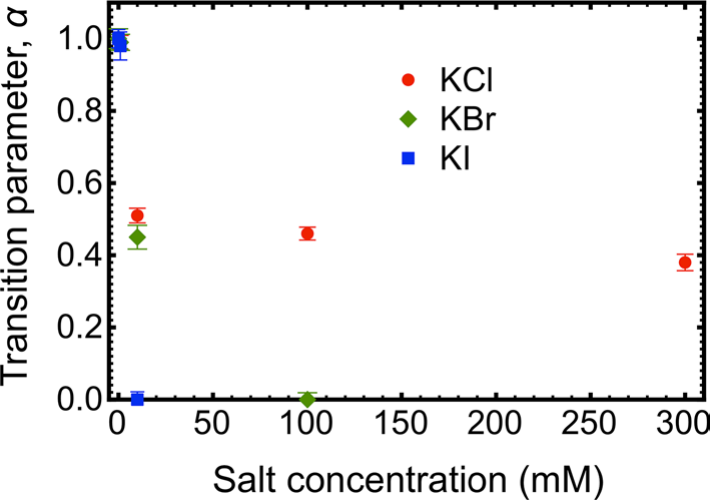
Salt concentration dependence of the transition parameter for the gold-coated tip in contact with a PDMAEMA brush in KCl, KBr, and KI solutions

The general transition Eq. (3) is a useful means of determining the balance between JKR and DMT behaviour. The transition parameter, *α*, is shown for the gold-coated tip interacting with the PDMAEMA brush layer in Fig. 6. The parameter is used to indicate whether the contact mechanics is best fitted with the JKR (*α* = 1), DMT (*α* = 0), or intermediate models. For the PDMAEMA brush-coated tip with the PDMAEMA brush layer, the transition parameter was measured to be *α* = 0.22 ± 0.01, 0.20 ± 0.01, and 0.19 ± 0.01 in 1 mM KCl, KBr, and KI, respectively. For KCl, *α* = 0 for greater concentrations, but for the other salts at the larger concentrations, the friction–load plot was linear. As the KCl concentration increases, the brush becomes less solvated, leading to an effective stiffening of the contact, while in KI the brush collapses completely giving rise to a transition to dissipation through ploughing. The ellipsometry data (Fig. 1) indicate that there is less of a collapse for KBr, but these results do not preclude any stiffening of the chain.

When the KCl concentration was increased from 1 mM to 300 mM, the transition parameter values (gold-coated tip) were intermediate between the DMT and JKR values (0 < *α* < 1) and they decreased gently with increasing salt concentration with a concomitant decrease in adhesion and the friction force (Figs. 4a, 5a). The frictional behaviour between the tip and the brush is in the region addressed by Maugis, whereby adhesive forces are effective even outside the area of contact, but the surface may be considered deformable [59].

When the PDMAEMA brush was probed by the gold-coated tip in KBr or KI solutions, a different behaviour to that in KCl solution was observed. The contact mechanics changes from JKR to DMT behaviour, followed by a linear friction–load relationship at 300 mM. For KI, the effect was more dramatic, with *α* = 0 for the KI solution at a salt concentration of 10 mM, and a linear friction–load behaviour at greater concentrations.

When PDMAEMA brush-coated AFM probes were used, a linear friction–load plot was observed for a high fraction of the salt solutions, suggesting that for these probes energy dissipation was predominantly through molecular ploughing. The relative friction coefficients for all samples are listed in Table 1. (The tabulated friction coefficients are relative to the most lubricious system, which is the brush–brush couple in a 300 mM KI solution.) The use of relative friction coefficients avoids the difficulties in comparing these results with a macroscopic coefficient of friction [60]. Linear (Amontons-like) behaviour was not observed for either tip coating at any salt concentration when KCl was used.

**Table 1:** Relative friction coefficients for gold- and PDMAEMA-coated tips in KI and KBr solutions (errors are less than 5%) determined from the slope of the friction–load plots

Salt concentration (mM)	Au		PDMAEMA	
	KBr	KI	KBr	KI
10			2.2	2.1
100		2.8	1.6	1.5
300	2.6	2.2	1.3	1.0

These coefficients are relative to that of the PDMAEMA brush-coated tip at 300 mM KI, which was set to unity

## Discussion

For all of the experiments performed in the present work, it is likely that the halide anions bind to the amine groups of PDMAEMA, with the level of binding following the Hofmeister series. Ellipsometry shows that the brush swells upon the addition of salt and remains constant up to 300, 100, and 10 mM for KCl, KBr, and KI solutions, respectively. The reduction in thickness suggests that the brush switches from an osmotic to a salted regime.

The reduction in adhesion (pull-off force) with increasing salt concentration may be a combination of two factors: the contact area and the tip–brush interaction, which includes both steric and electrostatic effects. The dominance of an electrostatic effect follows from the brush thickness remaining relatively constant for all KCl solutions, indicating that steric repulsion remains constant while the electrostatic interaction is screened. However, for large KBr and KI concentrations, the polymer brush takes a collapsed conformation with reduced steric repulsion, decreased contact area, and screened electrostatic forces. In such circumstances, the effect of a reduction in contact area is likely to have an important role in the contact mechanics.

There is a general tendency with the contact mechanics that increased salt concentration and heavier anions lead to a transition from JKR to DMT behaviour, and eventually a linear friction–load relationship. The single asperity contact mechanics on polymer brushes is dependent on both the magnitude of the tip–polymer interaction and the area of the contact that is determined by the solvation state of the polymer brush. The friction force measured could be the result of both effects. When the polymer brush layer is substantially swollen by the ions, it is susceptible to deformation, giving a large contact area. However, the binding of ions could screen attractive interactions between the AFM tip and the brush, and produce DMT mechanics or even a linear friction–load relationship, which suggests that the nature of the contact changes from soft and adhesive to stiff and less adhesive. The transition is supported by the adhesion results which show a decrease in adhesion with increasing salt concentration. In some cases, the friction–load relationship becomes linear, which suggests that energy dissipation is due to molecular ploughing. This is the case for both brush–gold and brush–brush interactions. The brush–brush couple is more lubricious and reduces the amount of salt required for ploughing to occur. Ploughing does not place specific requirements on the degree of solvation of the brush and has been observed for both collapsed and extended brushes [11]. A linear friction–load relationship was not observed for any salt at the concentrations used in this work for either experimental geometry. A similar phenomenon was reported in recent work on the effect of salt on the frictional properties of poly[2-(methacryloyloxy)-ethyl phosphorylcholine] (PMPC) which is highly lubricious and generally displayed a linear friction–load relationship [40]. In that particular study, KI was determined to have a larger coefficient of friction than KBr, but that is due to the nature of the interaction between halide anions and the functional groups.

Where a linear friction–load relationship was observed, relative friction coefficients were determined. The smallest coefficient was acquired in the 300 mM KI solution for the PDMAEMA brush-coated tip, while the largest was from the 100 mM KI for the gold-coated tip. Generally, the friction coefficient is smaller in KI solution than that in KBr for the same salt concentration and tip coating.

The impact of solvation state on the nanotribological characteristics has been demonstrated previously in a number of systems, including both self-assembled monolayers and polymer brushes [11, 39, 47]. The swelling of the PDMAEMA brushes governs the contact area with the AFM tip and hence the number of functional groups available to interact with the tip. It is therefore natural to expect JKR mechanics at low salt concentrations where the polymer brush is readily deformable.

The differences between the three halide anions examined in the present work on the tribological characteristics of the brushes are due to their affinity to the amine functional group of the PDMAEMA molecules, in agreement with the Hofmeister series. The most important factors on the enthalpy of the hydration for salt with water are the ion radius size and the ion charge, where an ion with a smaller radius (such as chloride) has greater hydration enthalpy than an ion with a larger radius (such as iodide) [61]. The amount of hydrogen bonding was found to reduce with smaller halide anions, as suggested by previous spectroscopic experiments [62]. It also confirms that iodide has stronger affinity to the amine groups, and is therefore more likely to be found close to the polymer chain and hence screen the brush charge more.

## Conclusions

The tribological properties of a polycationic brush, PDMAEMA, are dependent upon its salt environment and the nature of the anions. The smaller chloride anion has a significantly reduced effect on the contact mechanics and conformation (i.e. brush thickness) than the larger bromide and iodide anions. The addition of larger ions results in a reduced brush thickness in aqueous solution at high salt concentrations compared to that with no added salt. These larger ions also shield the adhesion between the tip and the brush. This is also the case when the tip is functionalized with PDMAEMA. The effect of the anions is best illustrated with the friction results, which demonstrate molecular ploughing for heavier ions, but JKR and DMT behaviour for lighter ions or at reduced salt concentrations. The most elastic system corresponds to a fully solvated brush in a water which exhibits pure JKR behaviour, and as salt is added, there is a transition to DMT contact mechanics. For the smallest anion (chloride), this transition is not achieved over the concentrations tested (up to 300 mM salt) and contact mechanics intermediate between DMT and JKR is observed. For the bromide and iodide, DMT behaviour is observed and, at greater salt concentrations, low adhesion and linear photodetector response is obtained, which suggests that molecular ploughing occurs.
